# Bilayer LDPC Codes Combined with Perturbed Decoding for MLC NAND Flash Memory

**DOI:** 10.3390/e26010054

**Published:** 2024-01-08

**Authors:** Lingjun Kong, Haiyang Liu, Wentao Hou, Chao Meng

**Affiliations:** 1 Faculty of Network and Telecommunication Engineering, Jinling Institute of Technology, Nanjing 211169, China; kong@jit.edu.cn (L.K.); mengchao@jit.edu.cn (C.M.); 2 Institute of Microelectronics, Chinese Academy of Sciences, Beijing 100029, China; 3 College of Telecommunications and Information Engineering, Nanjing University of Posts and Telecommunications, Nanjing 210003, China; 1220013235@njupt.edu.cn

**Keywords:** bilayer low-density parity-check (LDPC) codes, genetic algorithm (GA), perturbation noise, NAND flash memory, multi-level cell (MLC)

## Abstract

This paper presents a coding scheme based on bilayer low-density parity-check (LDPC) codes for multi-level cell (MLC) NAND flash memory. The main feature of the proposed scheme is that it exploits the asymmetric properties of an MLC flash channel and stores the extra parity-check bits in the lower page, which are activated only after the decoding failure of the upper page. To further improve the performance of the error correction, a perturbation process based on the genetic algorithm (GA) is incorporated into the decoding process of the proposed coding scheme, which can convert uncorrectable read sequences into error-correctable regions of the corresponding decoding space by introducing GA-trained noises. The perturbation decoding process is particularly efficient at low program-and-erase (P/E) cycle regions. The simulation results suggest that the proposed bilayer LDPC coding scheme can extend the lifetime of MLC NAND flash memory up to 10,000 P/E cycles. The proposed scheme can achieve a better balance between performance and complexity than traditional single LDPC coding schemes. All of these findings indicate that the proposed coding scheme is suitable for practical purposes in MLC NAND flash memory.

## 1. Introduction

In the past decade, NAND flash memories have been extensively applied in consumer electronic devices as they are characterized by nonvolatility, fast read and write speeds, and low power consumption. With the continuous miniaturization of the NAND process, novel storage methods—i.e., multi-level cell (MLC)/triple-level cell (TLC), and three-dimensional (3D) stacking technology—have been applied in order to improve the storage capacity of NAND flash memory [1]. These methods, however, inevitably cause a decrease in the storage reliability of NAND flash memories [2,3], which is characterized by lower program-and-erase (P/E) cycle endurance, increased susceptibility to cell-to-cell interference (CCI), and shorter data retention.

Error correction coding (ECC) is an effective technique for addressing the reliability problem of flash memories. In particular, low-density parity-check (LDPC) codes [4] and polar codes [5] have been applied in ultra-high-density flash memory systems due to their near-capacity performances in symmetric binary discrete memoryless channels (B-DMCs). The work of [6] provided a theoretical framework for constructing and analyzing LDPC codes for asymmetric storage channels. To tackle the asymmetric problem of MLC channels, the authors in [7] proposed an optimization algorithm based on reciprocal channel approximation (RCA) for extrinsic information transfer (EXIT) charts to optimize the degree distributions of an LDPC code, as well as for MLC read voltages. Due to their applications in rank modulation schemes for flash memories, codes in the $L_{1}$ metric have been widely investigated [8]. Two families of error-scrubbing codes based on the $L_{1}$ metric and a modular construction, which outperform conventional ECCs for MLC channels, were presented in [9]. According to intracell bit error characteristics and the error spacing strategy, the works of [10,11] improved the min-sum algorithm and bit-flip algorithm, respectively, for decoding LDPC codes (which can speed up the convergence and reduce the decoding delay). In our previous work [12], we designed new LDPC codes and rate-adaptive polar codes by utilizing the intrinsic properties of an MLC NAND flash channel. Specifically, the basis matrix of a protograph LDPC code was constructed based on a sequence of degrees that were optimized by the modified EXIT chart method for flash memories, while the rate-adaptive polar coding was achieved by iteratively calculating the new Bhattacharyya parameters of the memory cell bits. All of these findings suggested that the asymmetric property of errors among different pages can provide us with new insights for the design of ECC schemes for MLC NAND flash memories.

In the signal processing and coding areas, it can be shown that, under certain conditions, additional performance gains for detectors and decoders can be achieved by introducing noises to the received signals [13,14,15,16,17]. The authors in [16] presented a generalized framework and improved perturbation selection criteria for multi-round LDPC decoding schemes, in which pulse perturbations were applied to only a few symbols. A belief propagation list (BPL) algorithm that relies on artificial noise was proposed for decoding polar codes in [17]. The authors of [18] proposed a cyclic redundancy check (CRC)-assisted perturbation algorithm for decoding polar codes, wherbey when the CRC of a simplified successive cancellation (SSC) decoder fails, a number of possible candidate vectors for re-decoding are obtained by adding perturbation noises. In our previous work [19], we proposed several decoding algorithms for polar codes in AWGN channels, which can convert uncorrectable received sequences into the error-correcting regions of the sequences’ decoding space.

In this paper, we propose a coding scheme based on the bilayer LDPC codes in [20] for improving the reliability of an MLC NAND flash memory channel, where the channel model is the same as in [4]. Bilayer LDPC codes, an important class of structured LDPC codes used in cooperative relaying systems [21], can provide further diversity and coding gains by combining code words from a source code with the additional parity bits provided by a relay. In this work, we exploited the asymmetric error correction capabilities of MLC pages and placed the extra parity-check bits of a bilayer LDPC code in the lower page, which is a different approach from our previous work [12], where the differences betwene the memory cell bits were used to design ECC codes. These extra parity-check bits were activated only after the decoding failure of the upper page had occurred, which is where the channel state was relatively poor. Then, we applied the perturbation algorithm for the proposed scheme to further improve the decoding performance. The simulation results suggested that our proposed bilayer coding scheme can outperform the traditional LDPC coding scheme in MLC NAND flash channels. In addition, the proposed bilayer coding schemes can be easily extended to 3D flash memory.

Overall, the main contributions and novelties of this paper are summarized as follows:

1. A coding scheme based on bilayer LDPC codes is designed for an MLC NAND flash memory channel, which stores the extra parity-check bits in the lower pages with relatively good channel conditions. To the best of our knowledge, this is the first bilayer LDPC coding scheme that has been successfully designed for an MLC flash channel. The bilayer LDPC decoding algorithm is activated only when the decoding of the upper layer fails. Hence, the decoding complexity increases only slightly at low P/E cycle regions.
2. To further improve the decoding performance, the GA is applied to the proposed bilayer LDPC coding scheme, which produces the genetic noises that rapidly draw the received sequences back to their error-correctable decoding space. The reliability of the storage system is improved as a consequence.

The rest of the paper is organized as follows. In Section 2, we briefly review the related works on bilayer LDPC codes and the perturbation theory. In Section 3, the proposed bilayer LDPC coding schemes with perturbation decoding algorithms are presented. Section 4 presents the experimental results, and Section 5 concludes the paper.

## 2. Related Works

In this section, a brief review of related works is presented. We first provide an overview of bilayer LDPC codes. Then, we review the perturbation theory.

### 2.1. Bilayer LDPC Codes

**Figure 1:**
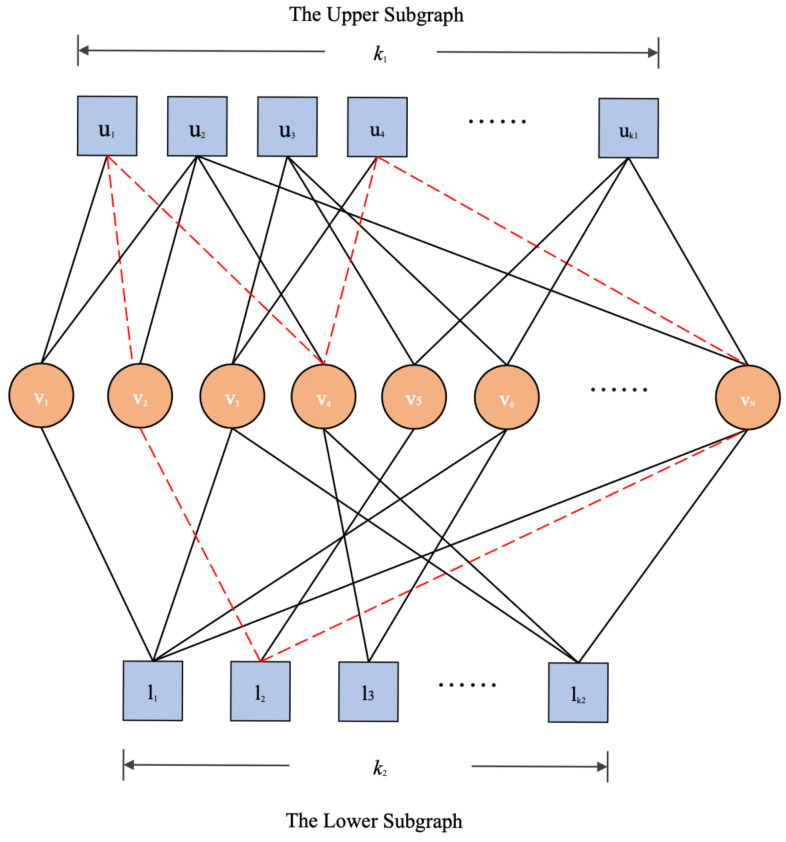
A Tanner graph for a bilayer LDPC code.

A binary bilayer LDPC code is represented by a Tanner graph, which is denoted by $\left(U_{\alpha }\cup V_{v}\cup L_{\beta },E\right)$, where $U_{\alpha }=\left\{u_{1},u_{2},\cdots ,u_{k_{1}}\right\}$ and $L_{\beta }=\left\{l_{1},l_{2},\cdots ,l_{k_{2}}\right\}$ are the sets of check nodes of the upper subgraph and lower subgraph, respectively; $V_{v}=\left\{v_{1},v_{2},\cdots ,v_{N}\right\}$ represents the set of variable nodes that connect the upper and lower subgraphs; and *E* is the set of edges such that $E\subseteq \left(U_{\alpha }\times V_{v}\right)\cup \left(V_{v}\times L_{\beta }\right)$ (see Figure 1). The red dotted edges in Figure 1 form a 6-cycle in bilayer Tanner graph that contains check nodes from both the upper subgraph and lower subgraph. The key to the construction of a bilayer LDPC code is how to jointly design the upper subgraph (the upper parity-check matrix $H_{u}$) and lower subgraph (the lower parity-check matrix $H_{l}$) so that both subcodes and the whole bilayer code have excellent error correction performances while maintaining low complexities.

The algebraic structures of the quasi-cyclic (QC)-LDPC codes facilitate their encoding and decoding processes, which leads to their wide applications [22]. Based on the index matrices that determine the shifts of the circulant matrices of parity-check matrices, QC-LDPC codes with a variety of code lengths and rates can be designed.

In this paper, we constructed bilayer QC-LDPC codes for MLC flash memory based on the design approach proposed in [20]. The parity-check matrices of QC-LDPC codes with various code rates can be constructed by selecting appropriate parameters for bilayer QC-LDPC codes. By following Theorem 1, as well as its corollaries in [20], we are able to design $H_{u}$ and $H_{l}$ jointly in order to ensure the constructed codes had good properties, e.g., large minimum and stopping distances. We refer the readers to [20] for further details on how to construct bilayer QC-LDPC codes.

### 2.2. Perturbation Theory

In this subsection, we will briefly introduce the perturbation theory. Benzi [23] discovered the stochastic resonance phenomenon, i.e., when the application of suitable noises can enhance the quality of a signal transmission [24,25]. The concept of stochastic perturbation opened up a new direction in the signal processing area. A perturbed decoding algorithm for a CRC-aided convolutional coding scheme was proposed in [26], which is where a perturbed received signal is decoded by the Viterbi algorithm when the CRC fails. The theory behind the perturbation decoding was analyzed extensively in [27]. As an inevitable interference factor in the storage process, the noise has a huge impact on information storage. Severe noise interference directly leads to the loss of stored information and affects the performance of the storage system. Based on the stochastic resonance phenomenon, artificially generating specific noise in the decoding space may compensate the effect caused by the channel noise and promote success in the decoding process [19].

**Figure 2:**
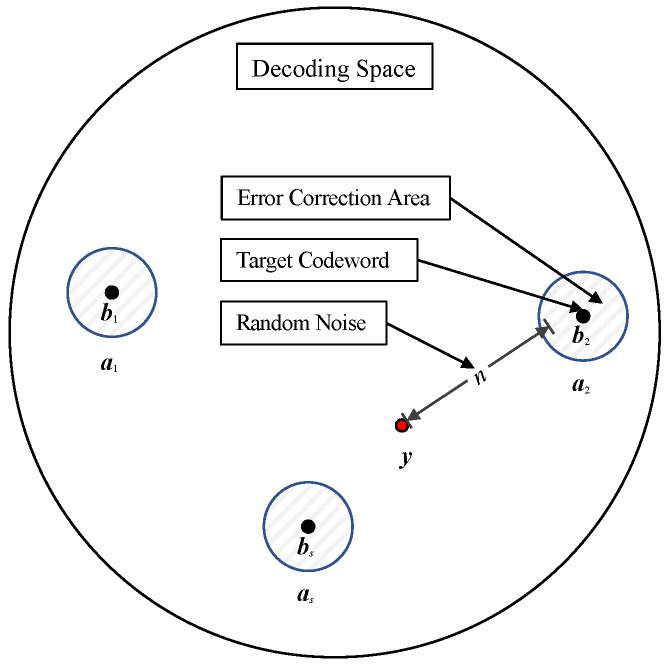
Schematic diagram of perturbation decoding.

Figure 2 illustrates the schematic diagram of the decoding space. Every valid code word $b\in \{b_{1},\;b_{2}\;,\ldots ,b_{s}\}$ in a decoding space has a corresponding error-correcting region $a\in \{a_{1},\;a_{2}\;,\ldots ,a_{s}\}$, where *s* represents the number of valid code words. The decoder can correctly decode when the signal y received is within the error-correcting region. Otherwise, the received signal is beyond the error-correcting area, thus indicating that the current channel noise influence has exceeded the error correction range of the decoder. If a stochastic perturbation noise n is added to a received signal that fails to be decoded, a new perturbation signal $y^{\prime }=y+n$ is constructed, which may fall into a certain error-correcting region and would then achieve a successful decoding.

However, the direction of random perturbation noise is arbitrary, and there is no guarantee that the noise can definitely help the received signal move to the correct error-correcting region. Therefore, some optimization algorithms can be used to artificially modify random perturbation noises, such that they can evolve in a direction that is helpful for correct decoding. Complex problems in fields like machine learning, combinatorial optimization, signal processing, and channel coding can be solved by the GA—a general framework that does not need gradient information or other auxiliary knowledge in the optimization process [28]. In particular, the GA has been widely used in the field of channel coding, such as in LDPC code construction and decoding [28,29], polar code construction and decoding [30,31], etc. It has been demonstrated in [19] that, in an AWGN channel, a reasonable selection of noise variance, as well as the evolutionary criterion, can obtain the desired perturbation noise and thus improve the decoding performance. In the following section, a GA-based perturbed decoding algorithm is proposed for the application of bilayer LDPC codes in an MLC flash channel.

## 3. Bilayer LDPC Codes Applied with a Perturbed Decoding Algorithm

In this paper, QC-LDPC codes were used as subcodes in a bilayer coding scheme that was applied to MLC flash channels. Then, a perturbed decoding algorithm for bilayer LDPC codes in MLC channels was proposed based on the perturbation principle.

### 3.1. Bilayer LDPC Code Design for an MLC Flash Channel

Assume that the code word begins with the information bits. The information sequence $c^{u}\;=\;\left[c_{1},c_{2},\ldots ,c_{N-k_{1}}\right]$ is encoded by the upper parity-check matrix, which is expressed by

(1) $$H_{u}\cdot x=H_{u}\cdot {\left[c_{1},c_{2},\ldots ,c_{N-k_{1}},p_{1},p_{2},\ldots ,p_{k_{1}}\right]}^{T}=0,$$

where $\left[p_{1},p_{2},\ldots ,p_{k_{1}}\right]$ contains the parity-check bits generated in the upper layer.

Suppose that the bilayer LDPC code’s upper parity-check matrix $H_{u}$ can be split into two submatrices as follows:

(2) $$H_{u}=[H_{u}^{\theta }|{\;}_{k_{1}\times \left(N-k_{1}\right)}|H_{u}^{\gamma }\left|{\;}_{k_{1}\times k_{1}}\right].$$

If $H_{u}^{\gamma }$ is a non-singular matrix, the $k_{1}$ parity-check bits can be obtained as follows:

(3) $${\left[p_{1},p_{2},\ldots ,p_{k_{1}}\right]}^{T}={\left[H_{u}^{\gamma }\right]}^{-1}H_{u}^{\theta }{\left[c_{1},c_{2},\ldots ,c_{N-k_{1}}\right]}^{T}.$$

The $k_{2}$ parity-check parity bits generated by the lower subgraph of a bilayer LDPC code (with a parity-check matrix $H_{l}$) are as follows:

(4) $$S={\left[q_{1},q_{2},\ldots ,q_{k_{2}}\right]}^{T}=H_{l}{|}_{k_{2}\times N}\cdot x.$$

The overall parity-check matrix is

(5) $$H=\left[\begin{array}{cc} H_{u}{|}_{k_{1}\times N} & {O|}_{k_{1}\times k_{2}}\\ H_{l}{|}_{k_{2}\times N} & {I|}_{k_{2}\times k_{2}} \end{array}\right],$$

and we have

(6) $$H\cdot \left[\begin{array}{c} X|{\;}_{N\times 1}\\ S|{\;}_{k_{2}\times 1} \end{array}\right]=\left[\begin{array}{c} 0|{\;}_{k_{1}\times 1}\\ 0|{\;}_{k_{2}\times 1} \end{array}\right].$$

For an MLC flash memory cell, the most significant bit (MSB) and the least significant bit (LSB) belong to an upper page and a lower page, respectively. The upper page bit and the lower page bit of the same cell are combined into one code word [4]. The bit error probabilities in flash cells are quite unbalanced, thus resulting in the unequal error rate performances of its upper and lower pages. In general, the raw error rate performance of the lower page is better than that of the upper page as the P/E cycle number increases [10].

**Figure 3:**
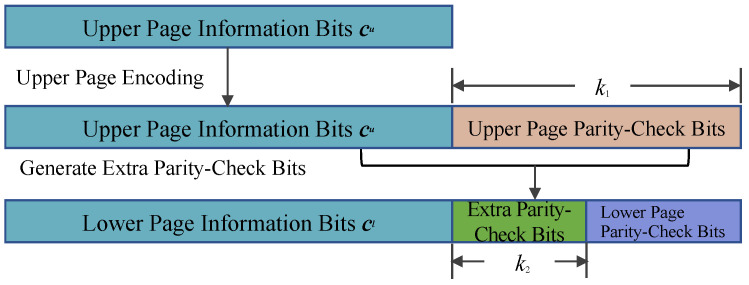
Schematic diagram of the bilayer LDPC encoding process in an MLC NAND flash channel.

Motivated by the observation of intracell unbalanced bit error probabilities in an MLC NAND flash channel, we proposed a coding scheme based on bilayer LDPC codes to improve the reliability and effectiveness of MLC flash memory systems. The specific encoding process established is as follows:

1. Upper Page Encoding: The upper page information sequence $c^{u}$ is encoded using the matrix $H_{u}$ to obtain the parity-check vector of length $k_{1}$ of the upper page from Equation (3).
2. Generation of the Extra Parity-Check Bits: Specifically, the upper page information bits and the corresponding parity-check bits generated in Step 1 are jointly encoded using the matrix $H_{l}$ to obtain the extra parity-check bits of length $k_{2}$, which are stored on the lower page.
3. Lower Page Encoding: Jointly encode the lower page information sequence $c^{l}$ and the extra parity-check bits of length $k_{2}$ from the upper page to generate the lower page parity-check bits via a separate channel coding scheme (which is not within the scope of this paper).
4. MLC Programming: The corresponding lower and upper bits are converted into voltages through an iterative incremental pulse programming method. The details of the bilayer LDPC encoding processes are shown in Figure 3.

**Figure 4:**
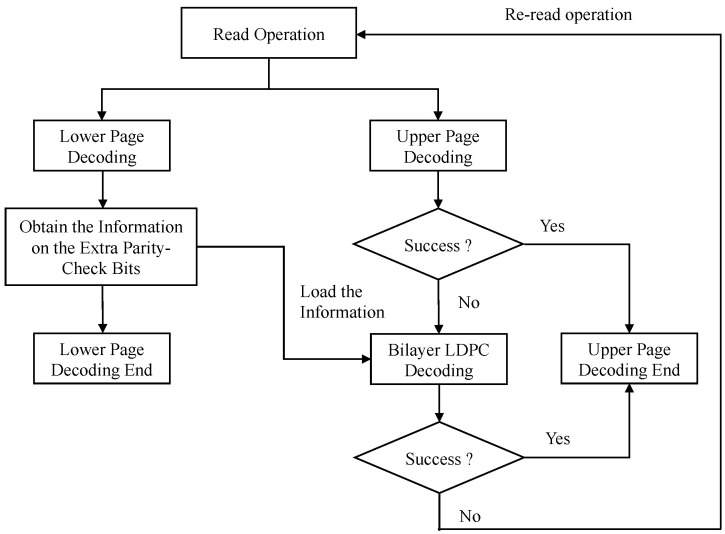
A flow chart of the bilayer LDPC decoding process in an MLC NAND flash channel.

In our bilayer LDPC decoding algorithm, the lower and upper pages are decoded separately, as shown in Figure 4. It is only when the upper page decoding fails that the information on the extra parity-check bits in the MLC lower page load and the bilayer LDPC decoding is performed. The specific decoding process is as follows:

1. Read Operation: The voltage in the MLC flash memory is quantified using the soft sensing method. We used the soft-decision storage sensing approach, in which more than one quantization level is used between two adjacent storage states. Thus, the soft information of the code word corresponding to the threshold voltage of each memory cell is obtained.
2. Lower Page Decoding: The lower page information bits and the extra parity-check bits of length $k_{2}$ are estimated. The information on the extra parity-check bits is stored for use in the bilayer LDPC decoding.
3. Upper Page Decoding: The upper page is decoded using the matrix $H_{u}$. If the decoding is successful, the decoding result is output and the decoding process is finished; otherwise, the information on the extra parity-check bits is read from the lower page and the bilayer decoding is carried out in the next step.
4. Bilayer Decoding: The bilayer decoding is performed until the decoding is successful or re-decoded with high-precision quantization.

The details of the proposed decoding algorithm of the bilayer LDPC code applied in an MLC channel are given in Figure 4. Note that, during the whole decoding process, the lower page decoding is employed only when the upper page decoding fails.

### 3.2. GA-Based Perturbed Decoding Algorithm

**Figure 5:**
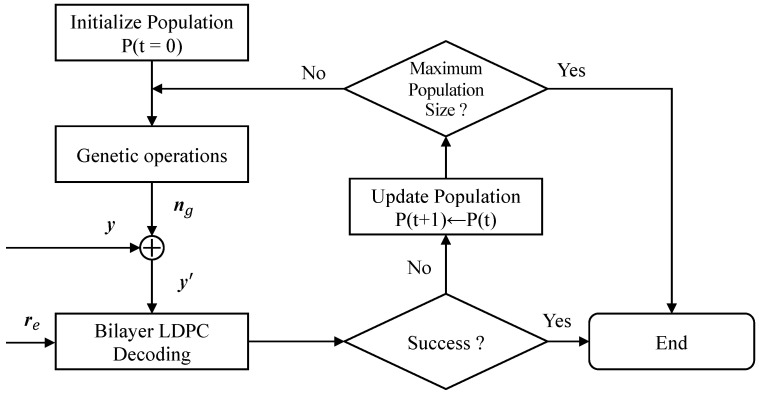
Block diagram of the perturbed decoding algorithm for a bilayer LDPC code.

In order to further improve the decoding performance in an MLC flash memory channel, a perturbed decoding method based on the GA was proposed. The proposed algorithm, on the premise of making full use of the asymmetric characteristics of an MLC channel, can reduce the number of extra parity-check bits required while ensuring a good decoding performance. The difference in this approach compared to our previous work [19] is that the algorithm proposed in this paper uses only the GA to generate the noises directly to assist the proposed bilayer LDPC coding scheme in an MLC NAND flash memory channel. As shown in Figure 5, when the decoding of a bilayer LDPC code fails, the perturbed decoding algorithm is activated. Then, the genetic noise generated by the GA will be added to the upper page bits for decoding again. The specific steps of the method are as follows:

1. Initialization: In the initialization process, a group of noises with Gaussian distribution is randomly generated by the noise generator as the initialization population. Compared with the channel noise, the artificially added perturbation noise power can be neglected. Genetic noise with a standard deviation between 0 and 0.385 is usually used. It should be noted that the length of the perturbed noise sequence is the same as that of the received sequence, which conforms to the coded form of the population. Hence, no additional coding operation is required for the population.
2. Crossover and mutation: By generating new individuals, the crossover and mutation operations expand the scope of the solution space and increase the diversity of the individuals—which are provided in the form of the ‘crossover’ function and ’mutation’ function, respectively—in Algorithm 1. Suppose two parents have chromosomes represented by $v=(v_{1},v_{2},...,v_{N})$ and $w=(w_{1},w_{2},...,w_{N})$, respectively, then—via crossover manipulation—the related children can be represented as
  (7) $$v_{c}\left[j\right]=\left\{\begin{array}{cc} v[j], & j\leq k\\ w[j], & j>k, \end{array}\right.$$
  (8) $$w_{c}\left[j\right]=\left\{\begin{array}{cc} w[j], & j\leq k\\ v[j], & j>k, \end{array}\right.$$
  where *k* is the crossover point, and *j* is an integer between 1 and *N*. Suppose an individual’s chromosome is represented by $v=(v_{1},v_{2},...,v_{N})$, and that the mutation probability is $p_{m}$, then the mutated chromosome $v^{\prime }$ can be created as follows:
  (9) $$v_{j}^{\prime }=\left\{\begin{array}{cc} v_{j}, & \mathrm{with}\;\mathrm{probability}\;1-p_{m}\\ \mathrm{random}\;\mathrm{value}, & \mathrm{with}\;\mathrm{probability}\;p_{m}, \end{array}\right.$$
  where *j* is an integer between 1 and *N*, and each gene has an equal probability of being mutated. In this paper, to simplify these operations, the individuals are randomly paired for a single-point crossover during the crossover process.
3. Fitness assessment and selection: A fitness evaluation is the only basis for the GA to update the population. The population’s fitness value is calculated by the fitness function, and the next generation is reproduced based on the fitness probability. An individual *i*’s fitness score is computed by
  (10) $$f_{c}^{\left(i\right)}=1\;/\left(1+\sum_{j=1}^{k_{1}+k_{2}}s_{j}\right)=1\;/\left(1+\sum_{j=1}^{k_{1}+k_{2}}H_{j}\;\cdot \;{\hat{y}}^{T}\right),$$
  where $H_{j}$ is the *j*-th row of the overall parity-check matrix of the bilayer LDPC code, and $\hat{y}$ is the hard decision result of the bilayer LDPC decoder (which corresponds to the received signal y). In the population update process, the fitness value of the population gradually increases as $\sum s_{i}$ decreases, and the probability $P^{\left(i\right)}$ of individual *i* being selected is obtained by the roulette wheel selection of Equation (11), which is provided in the form of the ‘selective’ function in Algorithm 1. When the decoding is correct, the individual has the highest probability of being selected, and this individual is the optimal in the perturbation decoding algorithm at that time. The following Algorithm 1 shows the details of the perturbed decoding algorithm:
  (11) $$P^{\left(i\right)}=\frac{f_{c}^{\left(i\right)}}{\sum_{j=1}^{M}f_{c}^{\left(j\right)}},$$
  where *M* is the number of individuals in the population.

**Algorithm 1:** Perturbed decoding algorithm.Input: y          // The received signal of the upper page           H        // Parity-check matrix of the bilayer LDPC code           *M*         // Population size           *T*          // Maximum number of iterations           $n_{g}$       // Genetic noise           $P_{g}$       // Population           $p_{c}$        // Crossover probability           $p_{m}$      // Mutation probability           $r_{e}$       // Extra parity-check bits from the lower page           $y^{\prime }$       // Perturbed bits Output: $\hat{x}$    // Estimated code word
1:Initialization: $\left.\hat{x}\leftarrow 0\right.$, $\left.t\leftarrow 0\right.$2:$\left.\hat{x}\leftarrow \mathrm{Bilayer}\;\mathrm{LDPC}\_\mathrm{decoder}(y,r_{e},H)\right.$3:Compute syndrome $s_{b}$ based on Equation (6)4:if $s_{b}==0$5:       break6:else7:    Initial population $P_{g}^{\left(0\right)}=\{n_{g}^{\left(i\right)}\left|i=1,2,\right.\ldots \left.,M\right\}$8:      while t<T9:                for i = 1 : 2 : M − 110:       $\left(n_{g}^{\left(i\right)},n_{g}^{\left(i+1\right)}\right)=crossover\left(n_{g}^{\left(i\right)},n_{g}^{\left(i+1\right)},p_{c}\right)$11:              end for12:                for i = 1 : M13:       $P_{g}^{\left(t\right)}=mutation\left(P_{g}^{\left(t-1\right)},p_{m}\right)$14:               end for15:                for i = 1 : M16:       $y^{\prime }=y+n_{g}^{\left(i\right)}$17:       $\left.\hat{x}\leftarrow \mathrm{Bilayer}\;\mathrm{LDPC}\_\mathrm{decoder}(y^{\prime },r_{e},H)\right.$18:       Compute syndrome $s_{b}$ based on Equation (6)19:       if $s_{b}==0$20:        break21:       end if22:               end for23:               Compute the individual fitness $f_{c}^{\left(t\right)}$ based on Equation (10)24:                $P_{g}^{\left(t\right)}=select\left(P_{g}^{\left(t-1\right)}\right)$25:                t←t+126:     end while27:end if28:Return $\hat{x}$

## 4. Simulation Results

The BER and FER performances of the bilayer LDPC coding scheme in an MLC NAND flash memory channel are evaluated in this section, where the P/E cycles directly reflect the channel model. For more details on the MLC NAND flash memory channel models, please see [4]. Unless otherwise mentioned, the performances given in this section are the experimental results on the upper page of an MLC. The parameters related to the encoding and decoding algorithms of the simulation are shown in Table 1.

### 4.1. Results of the Bilayer LDPC codes

**Figure 6:**
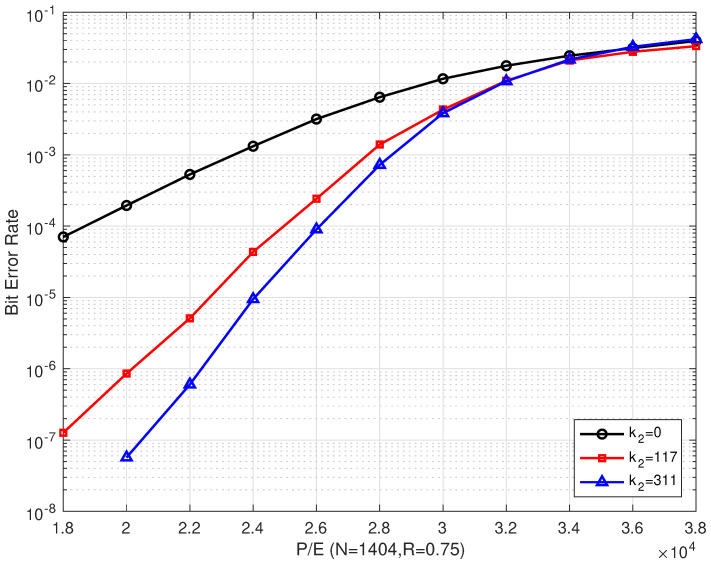
The BER performances of the proposed bilayer LDPC codes with different extra parity-check bits over different P/E cycles (where N=1404 and R=0.75).

**Figure 7:**
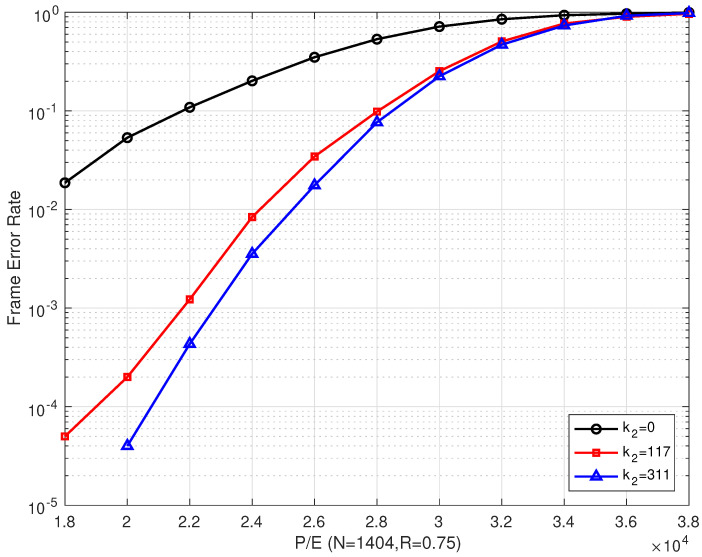
The FER performances of the proposed bilayer LDPC codes with different extra parity-check bits over different P/E cycles (N=1404 and R=0.75).

Figure 6 and Figure 7 illustrate the BER and FER performances of the proposed rate–0.75 bilayer LDPC codes with different coding schemes over different P/E cycles, respectively. Scheme I is decoded with only the upper LDPC code, where $k_{2}=0$ means no information on the extra parity-check bits (which is read from the lower page). Schemes II and III are decoded with bilayer LDPC codes, where $k_{2}=117$ and $k_{2}=311$ extra parity-check bits from the lower page, respectively. In addition, the lower page uses a separate LDPC code scheme with the same code length and code rate as the one used in the upper page. The extra parity-check bits received from the lower page are the decoding result of the lower page. In our simulations, the information bit length in the lower page was adjusted to obtain the number of extra parity-check bits, such that the code rate remained the same as that of the upper page.

Figure 6 shows that the proposed scheme outperformed the single LDPC code in Scheme I, where only the upper parity-check matrix $H_{u}$ was employed. Compared with Scheme I, the proposed bilayer LDPC codes in Schemes II and III extend the lifetime of MLC NAND flash memory by more than 7000 and 8000 P/E cycles at a BER of $10^{-4}$, respectively. Compared with Scheme I, the BER performance of the bilayer LDPC codes proposed in Schemes II and III can be enhanced by more than three and two orders of magnitude when P/E = 20,000, respectively.

In addition, the error correction performance of the bilayer LDPC codes is significantly enhanced as the number of extra parity-check bits increases. At a BER of $10^{-6}$, the bilayer LDPC codes proposed in Scheme II were able to extend the lifetime of the MLC NAND flash memory by more than 2000 P/E cycles when the number of extra parity-check bits was increased from $k_{2}=117$ to $k_{2}=311$. However, the bilayer LDPC codes suffered from a performance degradation as the P/E cycles increased. When the P/E was ≥ 34,000, the error correction performances of the three coding schemes were almost the same. This indicated that, in the final period of flash memory, even the enhanced coding scheme cannot improve its reliability.

Figure 7 shows that our proposed scheme outperforms Scheme I. This is consistent with the results in Figure 6. When P/E was ≥ 28,000, the error correction performance of Scheme II was only slightly better than that of Scheme III. Even with increasing the number of extra parity-check bits, the improvement in the error correction performance was limited.

**Figure 8:**
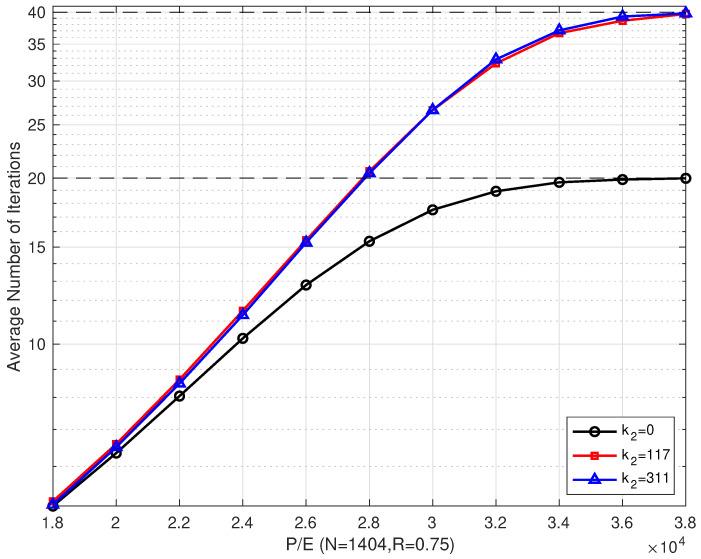
The average number of iterations of the proposed bilayer LDPC codes with different extra parity-check bits over different P/E cycles (N=1404 and R=0.75).

Figure 8 shows the comparison of the average number of iterations of the proposed schemes with different coding schemes that were implemented over different P/E cycles when R=0.75. When P/E was < 28,000, the average number of iterations of the proposed coding scheme was slightly higher than that of a single LDPC coding scheme, but it also brought a considerable gain in error correction performance. The reason for this was that the bilayer LDPC decoding algorithm was activated only after the upper page decoding failed. As the P/E cycle number increased, the channel condition of the upper page gradually became worse, and the number of bilayer LDPC decoding increased, which led to an increase in the number of iterations. In this situation, the proposed bilayer coding scheme could not improve the performance.

**Figure 9:**
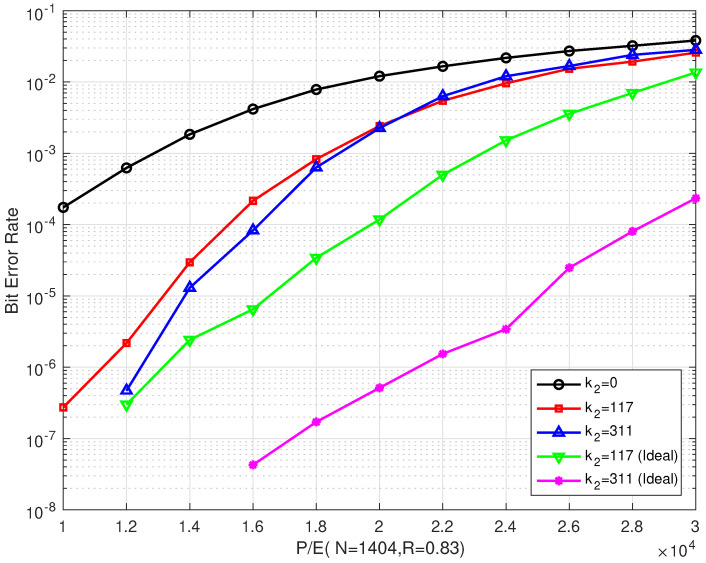
The BER performances of the proposed bilayer LDPC codes with different extra parity-check bits over different P/E cycles (N=1404 and R=0.83).

**Figure 10:**
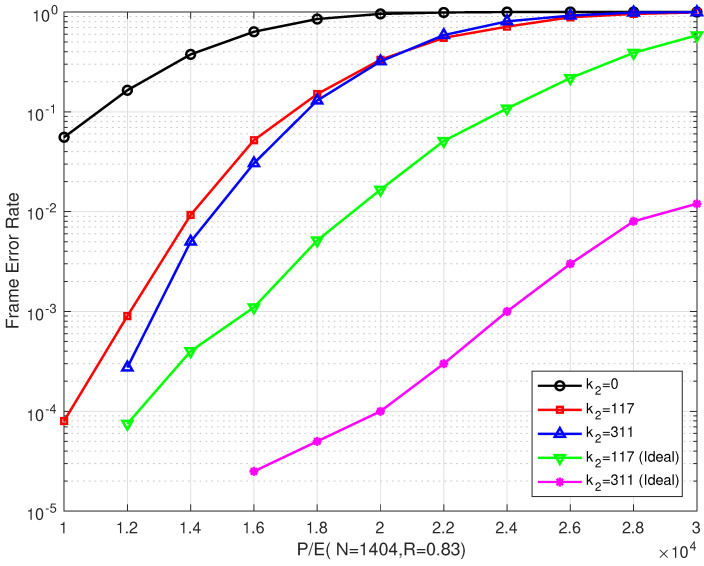
The FER performances of the proposed bilayer LDPC codes with different extra parity-check bits over different P/E cycles (N=1404 and R=0.83).

We also performed simulations on the rate–0.83 bilayer LDPC code, which is one of the proposed bilayer LDPC coding schemes described in Section 3, to further evaluate its error correction performance. The BER and FER performances of the different schemes are shown in Figure 9 and Figure 10, respectively. As the code rate increased, the proposed schemes outperform Scheme I in terms of error correction performance. To further confirm the importance of the extra parity-check bits from the lower page, we also present the performance of Scheme IV (green line) and Scheme V (purple line) in Figure 9 and Figure 10, respectively, for the ideal case (which assumes no errors in the extra parity-check bits).

We can also see from Figure 9 and Figure 10 that the proposed bilayer LDPC codes of Scheme IV and Scheme V can enhance the BER or FER performance by more than two and three orders of magnitude, respectively, compared with that of Scheme I when P/E = 12,000. Compared with Scheme II and Scheme III, the error correction performance of Scheme IV and Scheme V in the ideal case was extremely excellent, even in high P/E cycle regions. Compared with Schemes II and III, the BER or FER performance of the proposed bilayer LDPC codes of Schemes IV and V could be improved by more than one and three orders of magnitude, respectively, when P/E = 16,000.

From Figure 9, we know that the increase in the number of extra parity-check bits may not necessarily lead to a performance improvement with increasing the number of P/E cycles when R=0.83. This is because the wrong extra parity-check bits can cause an error propagation in the decoding process. In addition, the error propagation intensity may be reinforced with an increase in the number of parity-check bits. In this case, the extra parity-check bits become unnecessary since the decoding delay is increased but the error correction performance is not improved.

**Figure 11:**
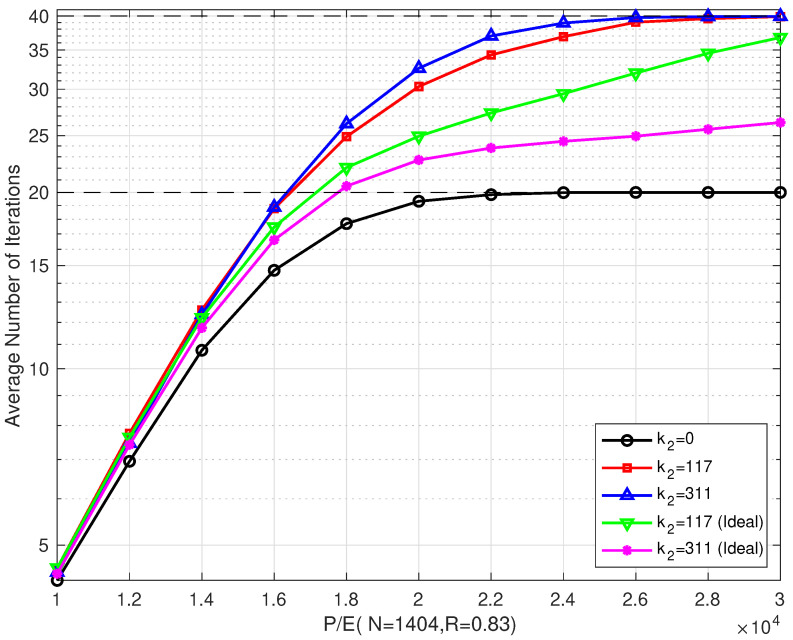
The average number of iterations of the proposed bilayer LDPC codes with different extra parity-check bits over different P/E cycles (N=1404 and R=0.83).

Figure 11 shows the comparison of the average number of iterations for several schemes in both ideal and non-ideal cases. We can see from the figure that the average number of iterations of the proposed several coding schemes is almost the same as that of the single LDPC coding scheme in low P/E cycle regions. However, as the P/E cycle number grew, the average number of iterations was higher than that of a single LDPC coding scheme. The average number of iterations of Scheme IV and Scheme V in the ideal case was lower than that in the non-ideal case. This confirms the fact that the errors in the extra parity-check bits can reduce the convergence speed and increase the decoding delay of the proposed method.

In addition, it can also be observed from these figures that the error correction performance improvement of the proposed scheme decreases as the code rate increases. This is because the error correction performance of the upper page becomes worse, and the errors in the extra parity-check bits exacerbate the deterioration.

### 4.2. Perturbed Decoding Algorithm Results

To evaluate the impact of the perturbed decoding algorithm on the performance of the proposed bilayer LDPC codes in MLC flash memory channels, computer simulations were performed. In the GA process, we set the crossover probability $p_{c}$ and the mutation probability $p_{m}$ to 0.8 and 0.05, respectively. The number *M* of the individuals in the population was set to 10. The number *T* of generations of the population updates was set to 5, and the standard deviation of the perturbed noise δ was set to 0.25.

**Figure 12:**
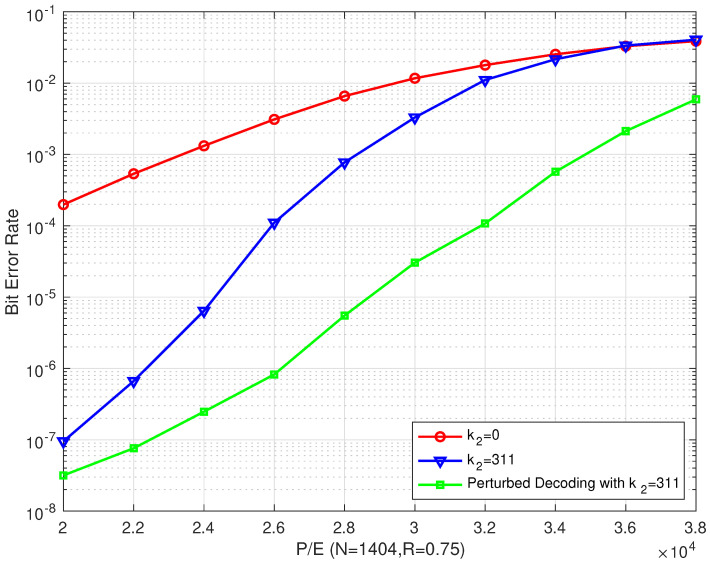
The BER performances of the proposed bilayer LDPC codes with perturbed decoding algorithm over different P/E cycles (N=1404 and R=0.75).

From Figure 12, we can see that the perturbed decoding algorithm outperformed other algorithms for our proposed bilayer LDPC codes. Compared with the conventional single LDPC decoding algorithm and the proposed bilayer LDPC decoding algorithm, the proposed perturbed decoding algorithm outperformed them by about 12,000 P/E cycles and 6000 P/E cycles, respectively, at a BER of $10^{-4}$. When P/E = 26,000, we can see from the figure that our proposed bilayer LDPC codes with perturbed decoding algorithm can achieve a significant improvement in the BER performance, which was found to be more than three orders of magnitude better than the conventional single LDPC decoding algorithm and two orders of magnitude better than the proposed bilayer LDPC decoding algorithm.

**Figure 13:**
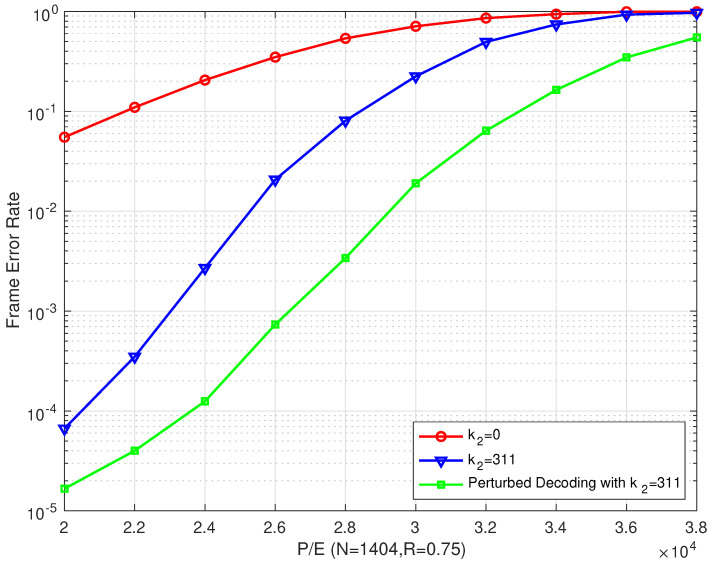
The FER performances of the proposed bilayer LDPC codes with perturbed decoding algorithm over different P/E cycles (N=1404 and R=0.75).

From Figure 13, we can draw the same conclusion that our proposed perturbed decoding algorithm for bilayer LDPC codes performs better than other algorithms. In addition, compared with the proposed bilayer LDPC decoding algorithm, an excellent error correction performance was achieved in both low and high P/E cycle regions.

**Figure 14:**
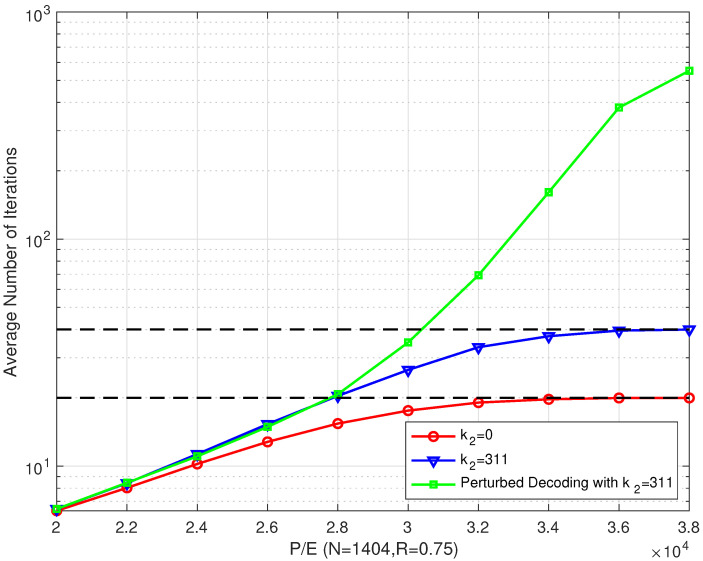
The average number of iterations of the proposed bilayer LDPC codes with a perturbed decoding algorithm over different P/E cycles (N=1404 and R=0.75).

As shown in Figure 14, the proposed bilayer LDPC decoding algorithm and the proposed perturbed decoding algorithm had the same average number of iterations. In addition, the conventional single LDPC decoding algorithm had a slightly lower average number of iterations. However, as the P/E cycle number increased, the gap between the numbers of iterations of the first two algorithms and the conventional single LDPC decoding algorithm gradually widened, especially when the P/E was larger than 30,000 cycles. When combining the above simulation results, we can see that, in low P/E cycle regions, the proposed perturbed decoding algorithm provides a good trade off between the performance and complexity. In high P/E cycle regions, the proposed perturbed decoding algorithm was still effective. However, it cost a large number of decoding iterations, which greatly increased the decoding delay.

## 5. Conclusions

In this paper, a coding scheme based on bilayer LDPC codes was proposed for MLC NAND flash channels by exploiting their intracell unbalanced bit error probabilities. When the conventional LDPC decoding of an upper page fails, the bilayer LDPC code is decoded with the extra parity-check bits of a lower page with better channel conditions, which can improve the reliability of MLC NAND flash systems. Compared with the conventional LDPC codes, the proposed bilayer LDPC codes significantly improve the BER/FER performance by more than three/two orders of magnitude. As the number of the extra parity-check bits increases, the bilayer LDPC codes can achieve a significant enhancement in error correction performance. According to the performance requirement of NAND flash memory, we can adjust the number of the extra parity-check bits of our proposed bilayer LDPC codes. In low P/E cycle regions, the proposed bilayer LDPC codes provide a considerable improvement in error correction performance at the cost of a slightly higher average number of iterations than single LDPC codes. In addition, we applied the perturbation theory to the proposed bilayer LDPC codes, as well as proposed a perturbed decoding algorithm for MLC NAND flash channels that can achieve a desirable error correction performance. In the future, we will try to design cost-effective coding schemes for high P/E cycle regions in high-density flash memory. In addition, we will explore combining the guessing random additive noise decoding (GRAND) [32] with the GA to take advantage of the asymmetric nature of the flash channels to improve decoding performance and reduce latency.

## Figures and Tables

**Table 1:** Simulation parameters of the bilayer LDPC codes.

Parameters	Values
Code word length *N*	1404
Length of extra parity-check bits $k_{2}$	0/117/311
Code rate *R*	0.75/0.83
Maximum number of BP iterations $I_{max}$	20

## Data Availability

The data presented in this study are available on request from the corresponding author.
